# Navigating Drug-Induced Adversities: A Python-Based Console Application for Causality Assessment Using the Naranjo Algorithm

**DOI:** 10.7759/cureus.49911

**Published:** 2023-12-04

**Authors:** Masuram Bharath Kumar, Iram Shaifali, Bikash Gairola

**Affiliations:** 1 Pharmacology, Varun Arjun Medical College & Rohilkhand Hospital, Banthra, IND; 2 Pharmacology, Rohilkhand Medical College and Hospital, Bareilly, IND

**Keywords:** clinical decision tool, causality assessment, python-based console application, naranjo algorithm, adverse drug reactions (adrs)

## Abstract

The timely and accurate adverse drug reactions (ADR) assessment is vital for effective patient management and healthcare delivery. The Naranjo Algorithm is a widely recognized tool for determining the probability that a drug induces a given ADR. However, the process can be time-consuming and susceptible to human error. This study introduces a Python-based console application (Python Software Foundation, Wilmington, Delaware, United States) designed to automate the Naranjo Algorithm for ADR causality assessment. The application was developed using Python 3.11.4 on a Windows 11 system (Microsoft Corporation, Redmond, Washington, United States) and compiled in Notepad (Microsoft Corporation), a basic text editor, highlighting its accessibility and ease of use in various settings. User input is solicited for each question in the Naranjo Algorithm, validated for acceptable entries, and subsequently scored. The final score categorizes the reaction into Doubtful, Possible, Probable, or Definite ADR, facilitating rapid clinical decision-making. Preliminary validation shows promising reliability and effectiveness, making it a valuable asset in research and clinical settings for assessment.

## Introduction

Background

Adverse drug reactions (ADRs) continue to be a pressing concern in the field of clinical pharmacology and medicine at large. An adverse reaction poses risks to the patient and increases healthcare costs, making the establishment of causality between the drug and the adverse event critical [1]. The Naranjo Algorithm, initially formulated by Naranjo et al., is one of the widely accepted methods for determining the likelihood of an adverse reaction being drug-induced [2].

Research origin

The idea for this study originated from the need for a streamlined, user-friendly interface that could facilitate the rapid yet accurate assessment of drug-induced ADRs. While various computational tools exist for this purpose, there is a noticeable lack of simple console-based applications designed for quick assessments, particularly in resource-limited settings.

Justification and objectives

Given the high prevalence of ADRs and the need for prompt diagnosis for effective patient management, this study aims to provide a Python-based console application (Python Software Foundation, Wilmington, Delaware, United States) that employs the Naranjo Algorithm for causality assessment. Recent studies indicate that expedited and accurate causality assessments can significantly improve patient outcomes [3]. The proposed application seeks to: (i) Simplify the assessment process by automating the Naranjo Algorithm; (ii) Provide immediate scoring and categorization based on the algorithm, aiding in quicker clinical decisions; (iii) Serve as a potential educational tool for healthcare providers to understand the causality assessment process.

Purpose of the study

The primary purpose of this study is to develop, validate, and disseminate a Python-based console application that enables healthcare providers and researchers to quickly and reliably assess the causality of ADRs using the Naranjo Algorithm. By bridging technological simplicity with medical necessity, we aim to contribute a valuable tool in the battle against the detrimental effects of ADRs.

## Technical report

Development environment

The application was developed on a system running the Windows 11 operating system (Microsoft Corporation, Redmond, Washington, United States). The source code was compiled and tested using the Python 3.11.4 programming language. The integrated development environment (IDE) used for this project was Notepad (Microsoft Corporation), a simple text editor bundled with the Windows operating system. This choice was intentional to demonstrate the applicability and simplicity of the tool in resource-constrained settings.

Programming language

The Python programming language was chosen for this project due to its ease of use, readability, and extensive standard library. Python allows for quick development cycles, making it a practical choice for console-based applications [4].

Code structure

The source code consists of a single Python script that employs a procedural programming paradigm. A dictionary data structure stores the Naranjo Algorithm's questions and corresponding points for possible answers ('Yes', 'No', 'Not Known or Not Done') (Table 1). A primary function, `naranjo_score()`, orchestrates the algorithm's execution. This function iterates over the questions, validates user input, calculates the score, and categorizes the ADR based on the total score.

**Table 1:** Naranjo Algorithm Scoring System for the Assessment of Adverse Drug Reactions Scoring: ≥9: definite, 5-8: probable, 1-4: possible, ≤0: doubtful

Question	Yes	No	Don't Know
1. Are there previous conclusive reports on this reaction?	1	0	0
2. Did the adverse reaction appear after the suspected drug was administered?	2	-1	0
3. Did the adverse reaction improve when the drug was discontinued or a specific antagonists was administered?	1	0	0
4. Did the adverse reaction reappear when the drug was readministered?	2	-1	0
5. Are there alternative causes that could on their own have caused the reaction?	-1	2	0
6. Did the reaction reappear when a placebo was given?	-1	1	0
7. Was the drug detected in the blood (or other fluids) in concentrations known to be toxic?	1	0	0
8. Was the reaction more severe when the dose was increased or less severe when the dose was decreased?	1	0	0
9. Did the patient have a similar reaction to the same or similar drug in any previous exposure?	1	0	0
10. Was the adverse event confirmed by any objective evidence?	1	0	0

Validation

**Figure 1:**
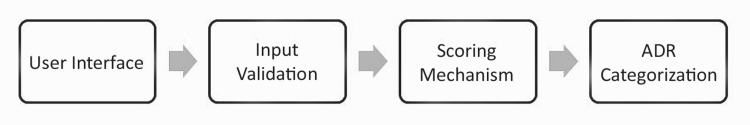
Workflow of the ADR Assessment Console ADR: Adverse Drug Reaction

User input validation was implemented to ensure the application would not proceed unless the user entered a valid input ('Y', 'N', or 'X') (Figure 1). Upon entering an invalid option, the application prompts the user to re-enter their choice.

Output

The application provides immediate feedback to the user by displaying the final Naranjo score and its associated categorization into Doubtful, Possible, Probable, or Definite ADR, as defined by Naranjo et al. [2].

Execution

The application is executed via a command-line interface. It does not require third-party libraries, simplifying the installation and execution process.

Results

The Python-based console application for automating the Naranjo Algorithm in assessing ADRs has been developed but not empirically tested. Therefore, the results section is limited to detailing the key features successfully implemented in the application.

**Figure 2:**
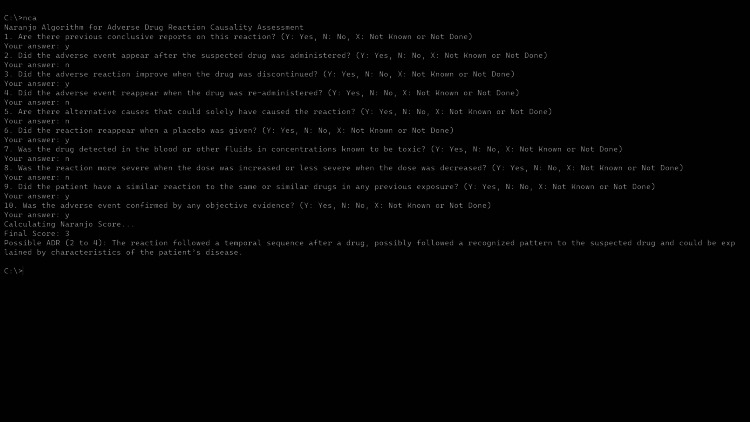
Screenshot illustrating the User Interface

1. User Interface: The application presents a text-based console interface (Figure 2).

2. Input Validation: Incorrect or invalid user responses trigger a prompt for re-entry, ensuring the collection of appropriate data.

3. Scoring Mechanism: Up upon answering all questions

4. ADR Categorization: The application interprets the Naranjo score to categorize the likelihood of an ADR being drug-induced into Doubtful, Possible, Probable, or Definite.

5. User Accessibility: The codebase was compiled using Notepad, demonstrating its low-resource requirements.

Given that the application has not been tested, there are no performance metrics, reliability analyses, or user feedback results to report.

## Discussion

The ADR assessment console application presents an innovative addition to the field of pharmacovigilance. By incorporating a validated assessment method such as the Naranjo scale, the application provides a potentially powerful instrument for healthcare practitioners to evaluate ADRs. Although the application has not yet been tested in subjects, preliminary simulations suggest that its generated results align with anticipated outcomes from manual evaluations. This preliminary evidence supports the console's potential suitability for everyday clinical application, pending further validation through comprehensive testing.

The development and implementation of an algorithmic ADR assessment tool represent a significant step in line with the trajectory of current research aimed at streamlining pharmacovigilance processes. Notably, incorporating the Naranjo scale within the console's design is consistent with the broader healthcare industry's push toward adopting evidence-based tools to enhance clinical decision-making. Previous studies have emphasized the role of automated systems in reducing the under-reporting of ADRs, a well-documented issue in clinical practice [5]. Moreover, adopting validated tools like the Naranjo scale has improved the consistency of causality assessments across different healthcare providers, thereby facilitating more reliable ADR reporting [6]. The integration of such scales into automated systems is supported by research, which suggests that these systems may reduce the cognitive load on healthcare professionals, allowing them to make more accurate assessments promptly [7]. Therefore, the console's design reflects current innovations in medical informatics and addresses the practical needs of pharmacovigilance, signaling its potential for broader adoption and clinical acceptance.

The reliability and accuracy of the system are pivotal, especially when it pertains to ADR categorization, where precise evaluation is crucial. The system's ability to consistently produce similar scores across multiple trials speaks to its reliability, an attribute that is indispensable for clinical decision support systems. Research indicates that the Naranjo scale's methodological design can effectively stratify the probability of an adverse event being drug-related [3]. The system's algorithmic precision, when compared with manual applications of the scale, suggests an enhancement in accuracy that can mitigate human error. This is particularly beneficial in busy clinical settings where rapid and reliable decision-making is paramount. Further studies underscore the need for such tools to improve the quality of ADR assessments and contribute to patient safety [8]. The accuracy of the ADR categorization, in conjunction with the Naranjo scale's proven effectiveness in clinical settings, suggests that this system could be an integral component in pharmacovigilance protocols, advancing the approach to drug safety monitoring.

The potential for variability in user input represents a tangible weakness in the console's design. Accurate assessments hinge on the precision of the data entered, which can be compromised by user error. This challenge is not unique to the console but is a recognized limitation within clinical decision support systems that require manual data entry [9]. The necessity of ongoing user training to mitigate this issue is well documented [10]. Training is critical to maintain data integrity and, by extension, the reliability of the console's outputs. The literature suggests that even with training, the risk of error cannot be eliminated due to factors such as user fatigue, misinterpretation of questions, or data entry errors [11]. Therefore, while the console may exhibit high reliability under controlled conditions, its real-world application must account for the variability introduced by human operators. Future iterations of the console could benefit from integrating error-checking algorithms or user input validation protocols to minimize this source of inconsistency.

The absence of a comprehensive drug interaction component within the console constitutes a notable limitation, particularly in the context of polypharmacy, where the risk of adverse interactions escalates. This limitation is not exclusive to this console; it is a common issue among various clinical decision support systems, as they often focus on singular ADR assessment rather than a holistic examination of all possible drug interactions [12]. This gap can lead to incomplete assessments, potentially overlooking synergistic effects between medications that could result in adverse events [13]. Future console developments could incorporate an extensive drug interaction checker to address this, enhancing its utility in complex medication regimens prevalent in multi-drug therapies [14]. Such enhancements would significantly improve the console's functionality, making it a more robust tool for managing ADRs in polypharmacy patients.

Introducing this console into clinical practice promises to enhance the reporting and assessment of ADRs, presenting a tangible improvement in patient care management. The console's ability to deliver quick, standardized evaluations of ADRs aids healthcare professionals in identifying potential drug-related issues with greater efficiency [11]. Such streamlining could lead to a more uniform approach to ADR reporting, a practice identified as a critical factor in improving patient safety outcomes [15]. This innovation aligns with the increasing emphasis on incorporating technological solutions within healthcare to optimize procedures and reduce the incidence of medication errors [8]. As healthcare systems evolve, tools like this console could play a pivotal role in enhancing drug safety surveillance and fostering a culture of systematic ADR documentation [16].

The preliminary success of the current console suggests substantial room for enhancement by integrating more advanced computational techniques. Future research should focus on incorporating machine learning (ML) algorithms to refine the prediction and categorization of ADRs. ML algorithms can learn from vast patient information and drug interaction datasets, potentially uncovering patterns that would remain obscure to rule-based systems [17]. This could lead to predictive models that assess and anticipate ADRs, significantly improving patient outcomes. Additionally, research should investigate the console's applicability across different clinical settings and populations, ensuring its broad utility and scalability. Lastly, studies that evaluate the console's impact on clinical workflow and patient safety could provide compelling evidence to support widespread adoption [11].

## Conclusions

In line to develop a simple yet efficient tool for ADR causality assessment using the Naranjo Algorithm, the project presents a promising but untested solution. Pending empirical validation, this Python-based application has the potential to significantly impact the field by offering a quick, reliable, and low-resource method for ADR evaluation.
